# Recent Advances in Shape-Controlled Synthesis of Noble Metal Nanoparticles by Radiolysis Route

**DOI:** 10.1186/s11671-016-1500-z

**Published:** 2016-06-10

**Authors:** Alam Abedini, Ahmad Ashrif A. Bakar, Farhad Larki, P. Susthitha Menon, Md. Shabiul Islam, Sahbudin Shaari

**Affiliations:** Institute of Microengineering and Nanoelectronics (IMEN), Universiti Kebangsaan Malaysia, 43600 Bangi, Selangor D.E. Malaysia; Department of Electrical, Electronic and System Engineering, Universiti Kebangsaan Malaysia, 43600 Bangi, Selangor D.E. Malaysia

**Keywords:** Noble metal nanoparticles, Gamma radiation, Shape controlling, Optical properties

## Abstract

This paper focuses on the recent advances on radiolysis-assisted shape-controlled synthesis of noble metal nanostructures. The techniques and protocols for producing desirable shapes of noble metal nanoparticles are discussed through introducing the critical parameters which can influence the nucleation and growth mechanisms. Nucleation rate plays a vital role on the crystallinity of seeds while growth rate of different seeds’ facets determines the final shape of resultant nanoparticles. Nucleation and growth rate both can be altered with factors such as absorbed dose, capping agents, and experimental environment condition to control the final shape. Remarkable physical and chemical properties of synthesized noble metal nanoparticles by controlled morphology have been systematically evaluated to fully explore their applications.

## Review

### Introduction

Recently, interest in metallic nanoparticles has been growing steadily due to their unique and fascinating properties and potential applications compared to their bulk counterparts [1, 2]. It is worth pointing out that behaviors of metallic nanoparticles strongly depend on the shape and size of particles [3, 4]. For instance, the computational and experimental studies of localized surface plasmon resonance (LSPR) and surface-enhanced Raman scattering (SERS) have revealed that the number, position, and intensity of LSPR modes, as well as the spectral region or polarization dependence for effective molecular detection via SERS, significantly vary with the shape and structure specially in the case of Au or Ag nanocrystals [2, 5, 6].

Many unusual properties that have been observed by shape variation arise as a result of the spatial confinement of electrons and phonons, their large surface-to-volume ratios, and electric fields around the nanoparticles [7–10]. These unusual properties make the anisotropic metal nanoparticles as promising structures for emerging applications in photonics, electronics, optical imaging, biomedical sensing, and electronic devices [1, 11–16]. Due to these reasons, more attention has been paid to find various parameters to control the morphologies and the size of nanomaterials and also will continue to be used as a tool for novel future applications.

As a result of strong plasmonic properties, noble metal nanoparticles have attracted great interest for many applications [17, 18]. In bottom-up synthesis approaches, this class of nanoparticles can be formed atom-by-atom in the gas phase, solid phase, or liquid phase [19]. The colloidal methods due to the ability to synthesize metallic nanoparticles directly in aqueous solutions have been extensively used [20, 21]. Since colloidal metal nanoparticles possess very active surface due to their high surface-to-volume ratio, protecting them from aggregation and controlling their shapes remain challenging. Therefore, multiple reaction parameters need to be carefully regulated in order to avoid heterogeneousity in size and shape of this class of nanoparticles. It has been shown that the method of preparation and specific experimental conditions can strongly modify their properties such as particles size, size distribution, morphology, and their stability [22, 23]. Among various techniques proposed for generation of noble metal nanoparticles in solution, the radiolytic method can be considered as one of the powerful techniques with various advantages. The radiolytically generated active species, such as hydrated electrons and transient radicals, exhibit strong reduction potentials which can reduce metal ions at each encounter [24–26]. Moreover, due to its ability in fine-tune dose of radiation, this method can offer good control over the morphology and distribution of metal nanoparticles [27].

This work is focused on the recent advances in the radiolytic synthesis of shape-controlled noble metal nanoparticles. In addition, several strategies on controlling the nucleation and growth process to obtain the shape-controlled metal nanoparticles and their remarkable properties will be reviewed.

### General Methods for Preparation of Metal Nanoparticles

The ideal techniques for preparation of metallic nanoparticles should be reproducible and possess ability to control the shape of the particles with monodispersity yields. Moreover, avoiding use of toxic precursors, using of environmentally friendly solvents, keeping the reaction temperature close to room temperature, and also minimizing the quantities of generated by-products are great advantages that make this method outstanding.

In bottom-up routs, most of the preparation techniques are based on the liquid phase category with the potential to synthesize large quantity of nanoparticles with good control of size, shape, morphology, crystallinity, composition, and surface chemistry at a reasonable low production cost [28]. Chemical method is the most frequently applied method for the preparation of metallic nanoparticles. This method can be categorized into chemical reduction methods [29, 30], electrochemical methods (electrolysis) [31], and radiochemical methods [32, 33]. Among these, radiochemical methods offer great advantages over other methods and particularly provide a clean technique for synthesis of noble metal nanoparticles.

#### Radiation Reduction Method

The synthesis of nanoparticles through radiation route requires an aqueous solution of metal salt, room temperature, and ambient pressure [34]. During radiolysis of water-hydrated electrons, $\left({\mathrm{e}}_{\mathrm{aq}}^{-}\right)$ and radicals such as $\overset{.}{\mathrm{H}},\;{}{}^{\cdot}\mathrm{O}\mathrm{H},{\mathrm{H}}^{+},{\mathrm{H}}_2{\mathrm{O}}_2,{\mathrm{H}}_2$ are produced [35]. The radiolysis of water is usually divided in three stages: physical stage (<10^−15^ s), physico-chemical stage (~10^−15^–10^−12^ s), and non-homogeneous chemical stage (~10^−12^–10^−6^ s) [36]. All mentioned active species and electrons are created in very high concentration at the end of the physico-chemical stage (Table 1).

**Table 1:** Different stages and their products in gamma radiation radiolysis of aqueous media [36]

Different stages of radiolysis of water	Important reactions
Physical stage (<10^−15^ s)	H_2_O ⇝ H_2_O^⋅ +^ + e^−^ H_2_O ⇝ H_2_O*
Physico-chemical stage (~10^−15^–10^−12^ s)	H_2_O^⋅ +^ + H_2_O → ^⋅^OH + H_3_O H_2_O* → H_2_O ${\mathrm{e}}^{-}\to {\mathrm{e}}_{\mathrm{th}}^{-}\to {\mathrm{e}}_{\mathrm{tr}}^{-}\to {\mathrm{e}}_{\mathrm{aq}}^{-}$
Non-homogeneous chemical stage (10^−12^–10^−6^ s)	${\mathrm{H}}_2\mathrm{O}\rightsquigarrow {\mathrm{e}}_{\mathrm{aq}}^{-},{\mathrm{H}}^{\cdot },{}{}^{\cdot}\mathrm{O}\mathrm{H},{\mathrm{H}}_2,{\mathrm{H}}_2{\mathrm{O}}_2,{\mathrm{H}}^{+},{\mathrm{O}\mathrm{H}}^{-}$

H_2_O^⋅ +^ is the ionized water molecule, H_2_O* is the excited water molecule, and sub-excitations electrons ${\mathrm{e}}_{\mathrm{th}}^{-}$ and ${\mathrm{e}}_{\mathrm{tr}}^{-}$ are thermalized and trapped electrons, respectively

Large number of hydrated electrons and H^•^ atoms which are produced during radiolysis of aqueous solutions are strong reducing agents with redox potentials of E^0^ (H_2_O/${\mathrm{e}}_{\mathrm{aq}}^{-}$) = −2.87 V_NHE_ and E^0^ (H^+^/H^•^) = −2.3 V_NHE_, respectively [28, 37]. Therefore, both can reduce monovalent or multivalent metal ions to state of zero-valent metal atoms through direct reaction or multistep process (Eqs. 1 and 2) [35].

1 $$ {\mathrm{M}}^{+}+{\mathrm{e}}_{\mathrm{aq}}^{-}\ \overset{\mathrm{Direct}\ \mathrm{reduction}}{\to }{\mathrm{M}}^0\;\mathrm{or}\;{\mathrm{M}}^{+}+\overset{.}{\mathrm{H}}\to {\mathrm{M}}^0+{\mathrm{H}}^{+} $$

2 $$ {\mathrm{M}}^{\mathrm{n}+}+{\mathrm{n}\mathrm{e}}_{\mathrm{aq}}^{-}\overset{\mathrm{M}\mathrm{ultistep}\ \mathrm{reduction}}{\to }\ {\mathrm{M}}^0\ \mathrm{or}\;\left({\mathrm{M}}^{\mathrm{n}+}+{\mathrm{e}}_{\mathrm{aq}}^{-}\to {\mathrm{M}}^{\left(\mathrm{n}-1\right)+},{\mathrm{M}}^{\left(\mathrm{n}-1\right)+}+{\mathrm{e}}_{\mathrm{aq}}^{-}\to {\mathrm{M}}^{\left(\mathrm{n}-2\right)+},\dots \right) $$

In contrast, sibling radicals which are also formed in radiolysis of water, such as ^•^OH, are able to oxidize the ions or the atoms into a higher oxidation state [38]. After appearing the zero-valent atoms or oxide particles, by cascade of coalescence processes, metallic or metal oxide nanoparticles will be formed. This part will be described comprehensively in the next section.

Radiolytically synthesized nanoparticles are in the form of colloidal particles which possess huge surface-to-volume ratio and high specific surface area [28]. As a result, a large part of surface of the particle atoms can be in contact with the surrounding liquid. This implies the formation of soluble macromolecules, which increase the rate of interactions or in another words fasten the reactions. Thus, colloidal nanoparticles are thermodynamically unstable, which in the absence of counteracting force will grow and colloidal system with nanoparticles in various shapes will be formed [39].

### Nucleation and Growth

In the synthesis of metallic nanoparticles in liquid-phase system, “nucleation” can be clarified as the formation of a small cluster from aggregation of newly formed atoms in the solution [40]. Due to the high binding energy between two metal atoms or atoms with unreduced ions, the newly formed neutral M^0^ atoms at first dimerize when encountering or being associated with the excess M^+^ ions (Eqs. 3 and 4).

3 $$ {\mathrm{M}}^0+{\mathrm{M}}^0\to {\mathrm{M}}_2^0 $$

4 $$ {\mathrm{M}}^0+{\mathrm{M}}^{+}\to {\mathrm{M}}_2^{+} $$

The charged dimer clusters (M^+^_2_) may further be reduced to form a center of cluster nucleation or leads to cluster growth. The strong bonding between clusters with unreduced ions (Eq. 5) or between two charged clusters leads to fasten association processes (Eq. 6).

5 $$ {\mathrm{nM}}^0+{\mathrm{x}\mathrm{M}}^{+}\overset{\mathrm{Ion}\ \mathrm{association}}{\to }{\mathrm{M}}_{\left(\mathrm{n}+\mathrm{x}\right)}^{\mathrm{x}+} $$

6 $$ {\mathrm{M}}_{\left(\mathrm{n}+\mathrm{x}\right)}^{\mathrm{x}+} + {\mathrm{M}}_{\left(\mathrm{m}+\mathrm{y}\right)}^{\mathrm{y}+}\overset{\kern1.25em \mathrm{Association}\ \mathrm{of}\ \mathrm{charged}\ \mathrm{cluster}\kern1em }{\to }\ {\mathrm{M}}_{\left(\mathrm{p}+\mathrm{z}\right)}^{\mathrm{z}+} $$

where *m*, *n*, and *p* represent the nuclearities and *x*, *y*, and *z* stand for number of associated ions.

**Fig. 1:**
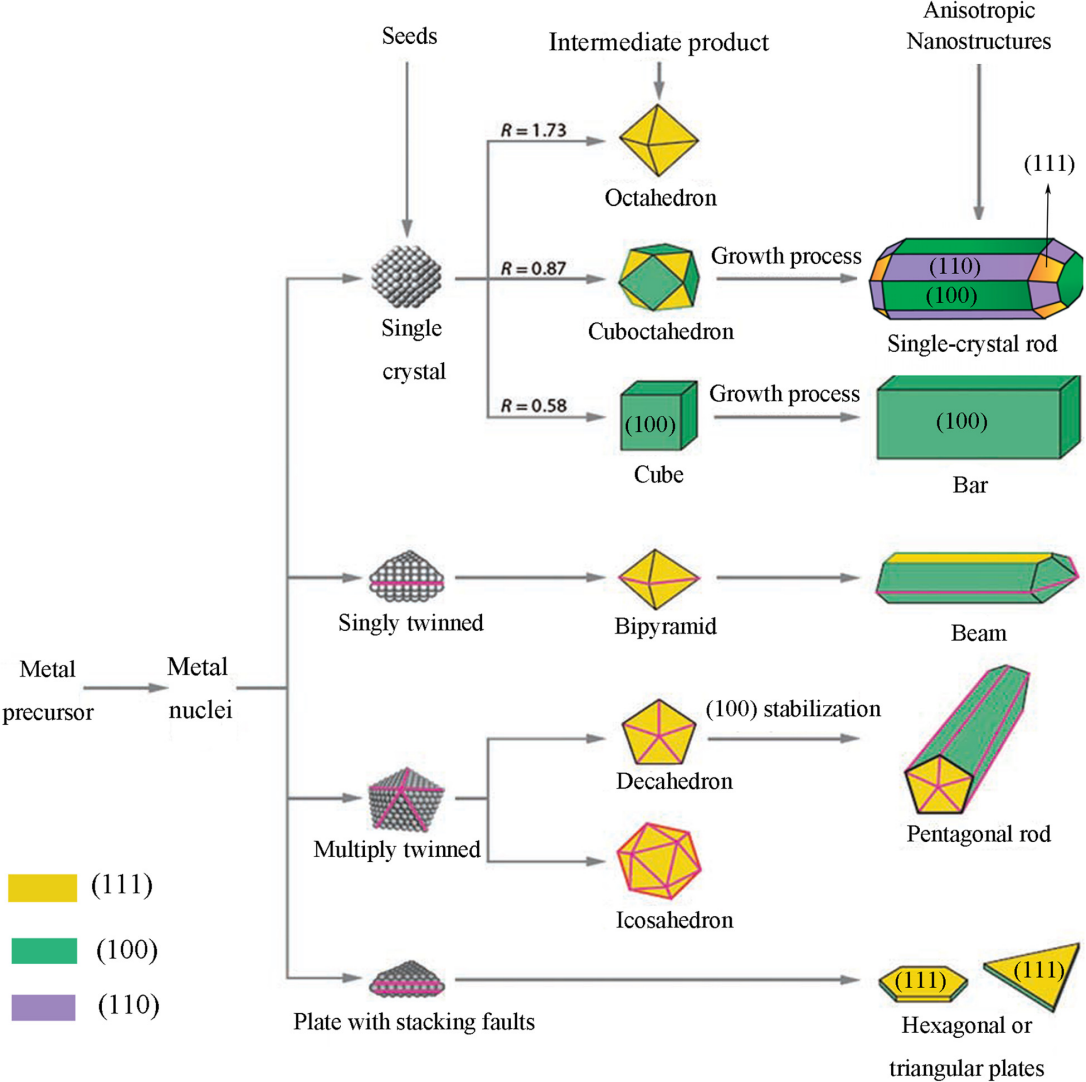
A schematic illustration of the reaction pathways that lead to formation of metal nanoparticles with different shapes. The ratio between the growth rates along the (100) and (111) directions parameter have been presented by R. (Original figure from references [9, 40])

These nuclei grow and past a critical size and seeds with several possible shapes will be formed. The crystallinity of seeds, which determined by the minimization of surface energy, plays the most important role in controlling the shape of final products [41]. Figure 1 schematically presents the formation of noble metal nanoparticles in different shapes with respect to the various stages of the reaction. As it can be seen, an octahedron, cuboctahedron, cube, or octagonal rod can be grown from a single crystal seed [42], whereas a decahedron, icosahedron, or pentagonal rod can grow from the multiply twinned particle [43, 44]. According to the Wulff facets theorem, a crystal has to be bounded by facets giving a minimum total surface energy at equilibrium [45, 46]. The surface energies corresponding to different facets in face-centered cubic (*fcc*) noble metals (e.g., Ag, Au, Cu) usually increase in the order of *a*_(111)_ < *a*_(100)_ < *a*_(110)_ [47, 48].

#### Strategies for Controlling the Shape under Gamma Irradiation

In the nano-sized regime, due to the high surface-to-volume ratio of particles, the thermodynamic and kinetic considerations for nanocrystal formation are more complicated compare to the bulk materials [49]. Therefore, overcoming this surface energy to control the morphology of nanoparticles still remains a challenge.

In order to predict the morphology of radiolytically synthesized nanoparticles, various methodologies have been recently developed. These strategies can be categorized in four different ways: (a) kinetically control the growth rates of various facets by using capping reagents and appropriate absorbed dose, (b) use of organic or inorganic templates to control the shape, (c) effect of anion, and (d) assembly of presynthesized nanoparticles. In the following, we will discuss these methods comprehensively as well as advantages and challenges associated with each one.

#### Growth Control by Using Capping Reagent and Absorbed Dose

Colloidal metallic nanoparticles, particularly in the absence of counteracting force, are attracted to each other by the van der Waals force [50, 51]. Consequently, overcoming this attractive force is one of the great challenges to control the morphology of nanoparticles. There are several types of capping agents to balance these attraction forces by electrostatic stabilization and/or steric stabilization. The type of capping agents depends on the kind of metal, expected morphology, and the application of the resultant nanoparticles.

**Fig. 2:**
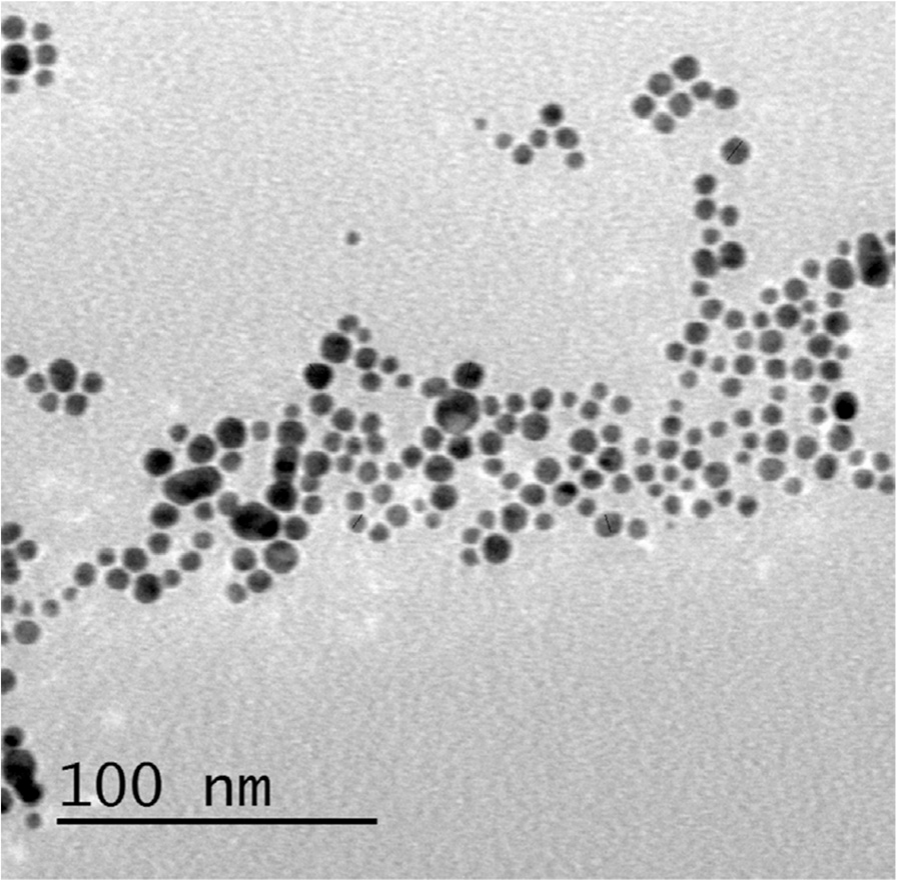
TEM image of spherical Au nanoparticles. TEM micrograph of the developed Au nanoparticles after gamma-irradiation reduction at 6MR(~53 kGy) [27]

The shape of colloidal particle mainly results from its surface energy, which is defined as the excess free energy per unit area for a particular crystallographic face in nanoscale. Homogeneous nucleation and seed growth under equilibrium conditions favor the formation of spherical objects [52]. There are several reports about the synthesis of spherical colloidal nanoparticles by gamma radiation routes [53, 54]. Figure 2 shows the typical TEM micrographs of spherical Au nanoparticles synthesized by gamma irradiation and using *Chenopodium murale* leaf capping agent [27].

The crystal morphology can also be considered in terms of growth kinetics, by which the fast growing facets have high surface energies and they will vanish to leave behind the slowest growing ones as the facets of the product. Therefore, by introducing appropriate capping reagent(s) to change the free energies of the various crystallographic facets and altering their growth rates, the final shape of a crystal can be controlled.

**Fig. 3:**
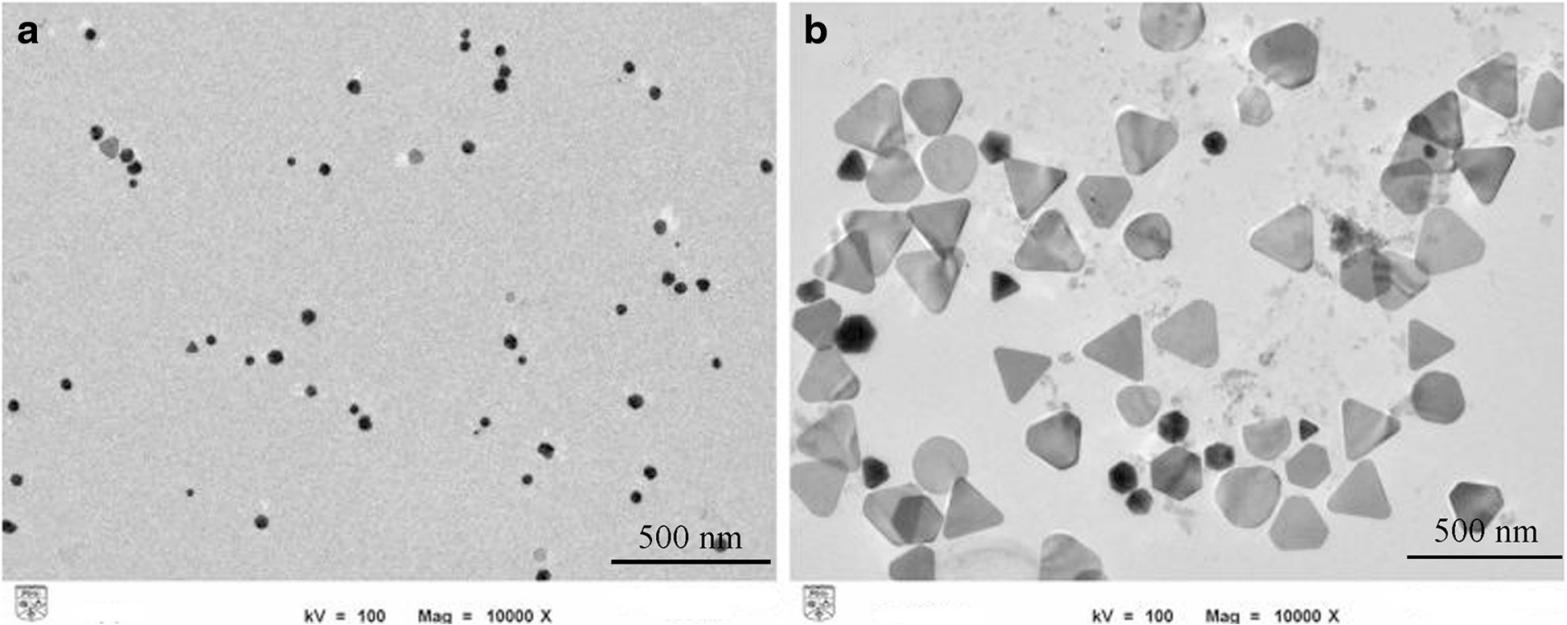
TEM images of irradiated samples of Ag colloids at different gamma dose. **a** At 30 kGy, resultant particles are mostly in the form of spherical. **b** At 100 kGy, triangular nanoplates are dominant [55]

During the radiolytic formation of nanoparticles, the rate of reduction and growth, which can be affected by varying the absorbed dose, influences the shape of final products [28]. For example, during the preparation of Ag nanoparticles by gamma irradiation in the presence of PVA at low irradiation doses, when the initial growth rate is slow, capping rate dominates and consequently small spherical nanoparticles are formed (Fig. 3a). By increasing gamma irradiation dose, rapid adsorption of the cross-linked PVA chains on the (111) facets of newly formed silver nanoparticles lead to the shape transition from spherical form to triangular nanoplates (Fig. 3b). On the other hand, at higher gamma dose, larger triangular nanoplates with (111) facets at flat top and bottom faces and (110) facets at edges will be formed since the reduction rate of Ag^+^ promotes anisotropic growth along the (110) facets [55].

**Fig. 4:**
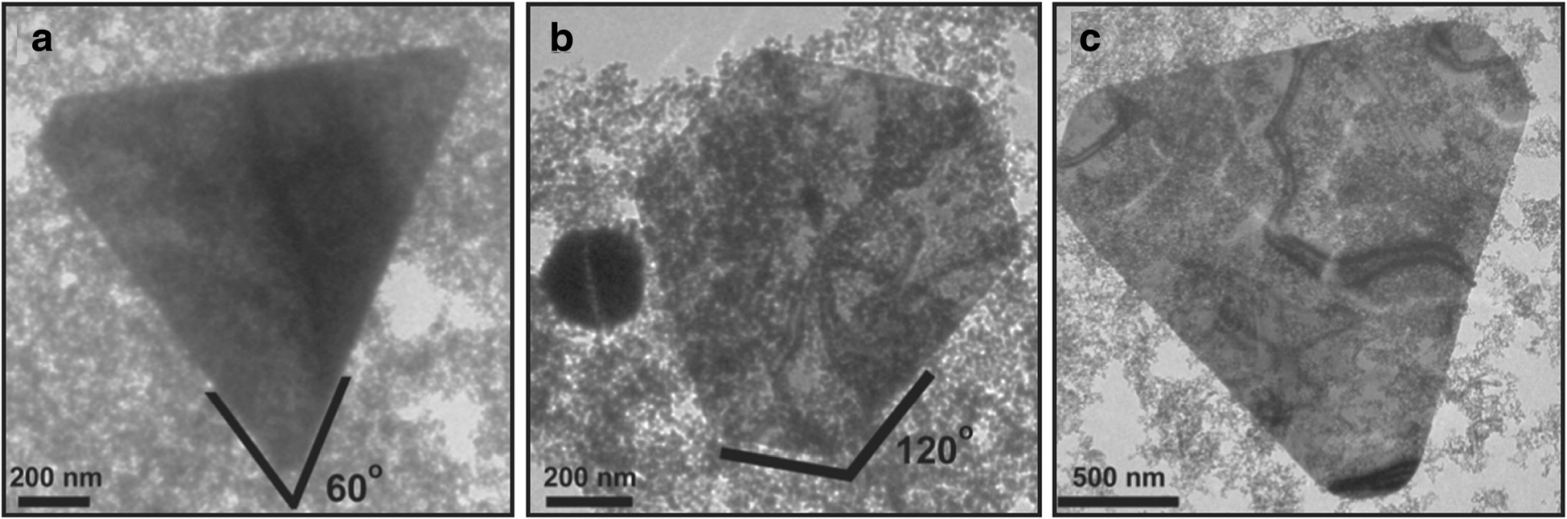
TEM images of gold nanoplates with anisotropic structures. **a** triangular, (**b**) hexagonal, and (**c**) truncated triangular shape [56]

Lou et al. [56] have reported radiolytic fabrication of single-crystal Au nanoprisms with anisotropic structure of triangular, hexagonal, and truncated triangular (Fig. 4). It was investigated that (3-aminopropyl) triethoxysilane (APTES)-coated Fe_3_O_4_ nanoparticles play important roles in the formation of Au nanoplates. Strong adsorption of the active amino group of the APTES-coated Fe_3_O_4_ on the selected facets of gold nuclei make this surface become hindered in growth process while the others grow fast. Finally, growth rate was reduced along the adsorbed surface (111) and enhance along (110) direction which can lead to the formation of anisotropic plate form of Au nanoparticles.

**Fig. 5:**
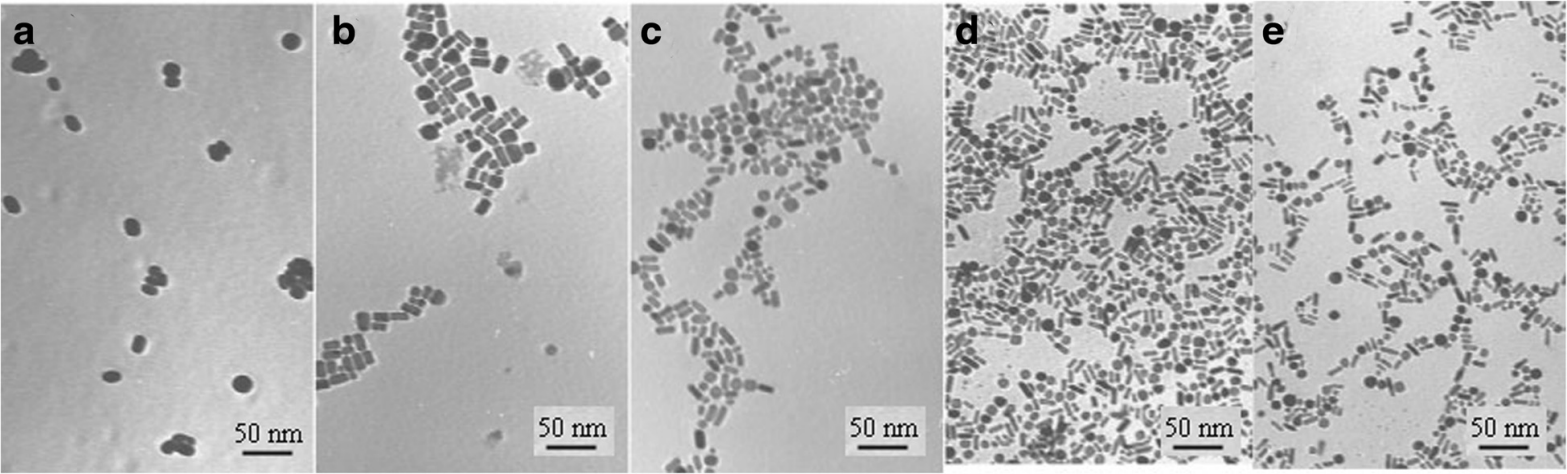
TEM images of Au nanoparticles. TEM images of Au nanoparticles synthesized by gamma-ray irradiation at different dose rate: **a** 1.0 kGy/h, **b** 3.0 kGy/h, **c** 6.0 kGy/h, **d** 10 kGy/h, and **e** 13.6 kGy/h [57]

Similar techniques, using different membranes, have also been successfully applied to the synthesis of Au nanorods by Okamoto et al. [57]. Au nanorods with larger aspect ratio are synthesized with the higher dose rate in the presence of cetyltrimethyl ammonium bromide (CTAB) as a capping agent (Fig. 5). At high dose rate, adsorption of CTAB molecules on (110) planes and, on the other hand, the fast reduction rate at the other facets caused an anisotropic growth and formed small size rod-shaped Au nanoparticles. At low dose rate, slow reduction rate leads to form the large nucleated seed crystals and also isotropic growth on all facets resulting in large size and the quasi-spherical Au nanoparticles.

#### Using of Organic and Inorganic Templates

**Fig. 6:**
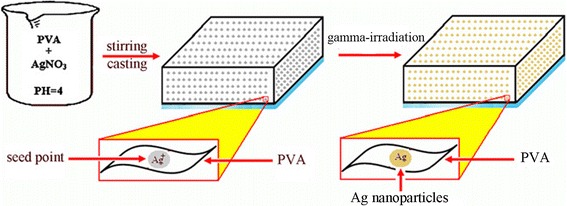
The process of seeded growth of Ag nanoparticles on PVA matrix [58]

During the formation of metal nanoparticles in bottom-up approach, rates of nuclear formation and growth can be controlled by choosing appropriate templates. These templates mostly possess nano-sized pores which can be used to fabricate shape-controlled metal nanoparticles. For example, the seeded growth of Ag nanoparticles within PVA matrix has been reported by Eisa et al. [58]. In this synthetic strategy, by preparation of metallopolymers films, metal centers are embedded directly into the polymer backbone (Fig. 6).

**Fig. 7:**
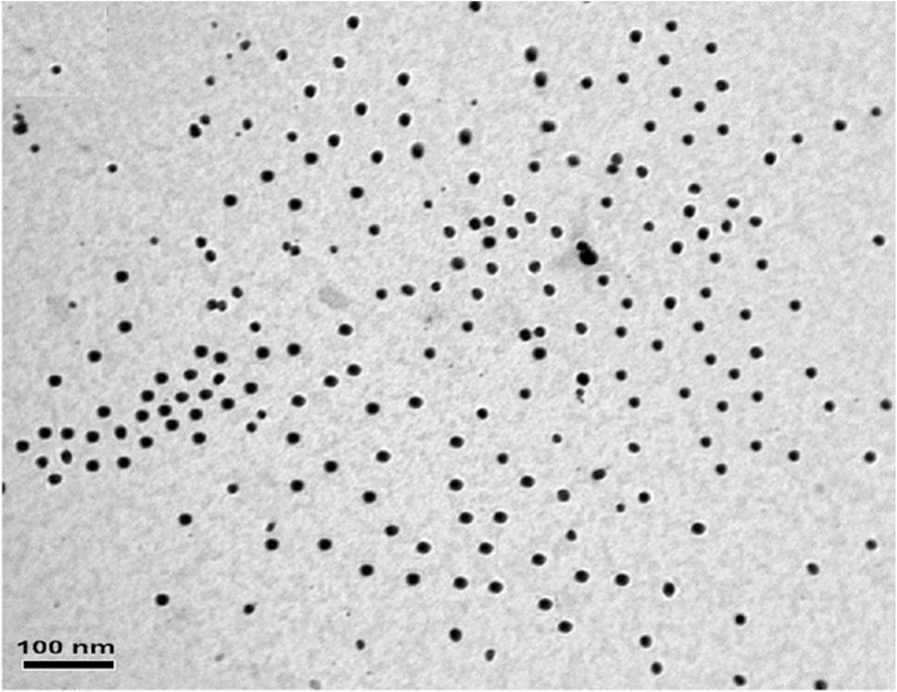
TEM image of spherical Ag nanoparticles. TEM image of resulted Ag nanoparticles for 75 kGy-irradiated PVA/Ag nanocomposites (adopted from [58])

At the cluster surface, ^⋅^OH groups of PVA molecule, which appear under gamma irradiation, anchor the Ag^+^ ion and reduce these ions to form of Ag^0^ atoms. Therefore, formation of silver nanoparticles can be attributed to the direct reduction between PVA and Ag^+^ ions [58]. The resultant nanoparticles are monodispersed spherical Ag nanoparticles (Fig. 7).

**Fig. 8:**
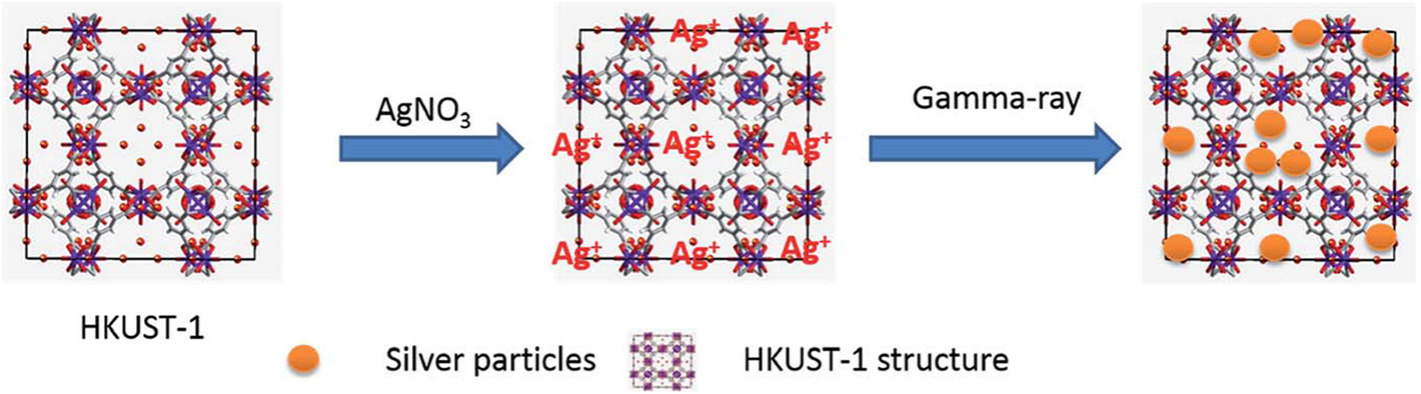
Schematic illustration on preparation of Ag@HKUST-1 crystals. (Adopted from [59])

Recently, radiolytic synthesis of ultrafine Ag nanoparticles by using HKUST-1 crystals as metal-organic framework (MOF) template has been reported [59]. MOF materials were used in order to prevent the aggregation of nanoparticles. Due to their special architectures, MOF materials provide well-defined interconnected pore channels, large internal surface areas, and tunable surface chemistry that make them as an appropriate template for synthesis of the nanoparticles [60–62]. HKUST-1 crystals (Cu^2+^ coordinated to trimesic acid (TMA) linkers) have been introduced as a hard template for Ag nanoparticle synthesis by He et al. [59]. The crystal structure of HKUST-1 is a face-centered cubic with large square shape which at the (111) direction of this cubic cell, there are large hexagonal-shaped windows in honeycomb arrangement. These confined cages formed very adequate cavities for growing nanoparticles (Fig. 8).

**Fig. 9:**
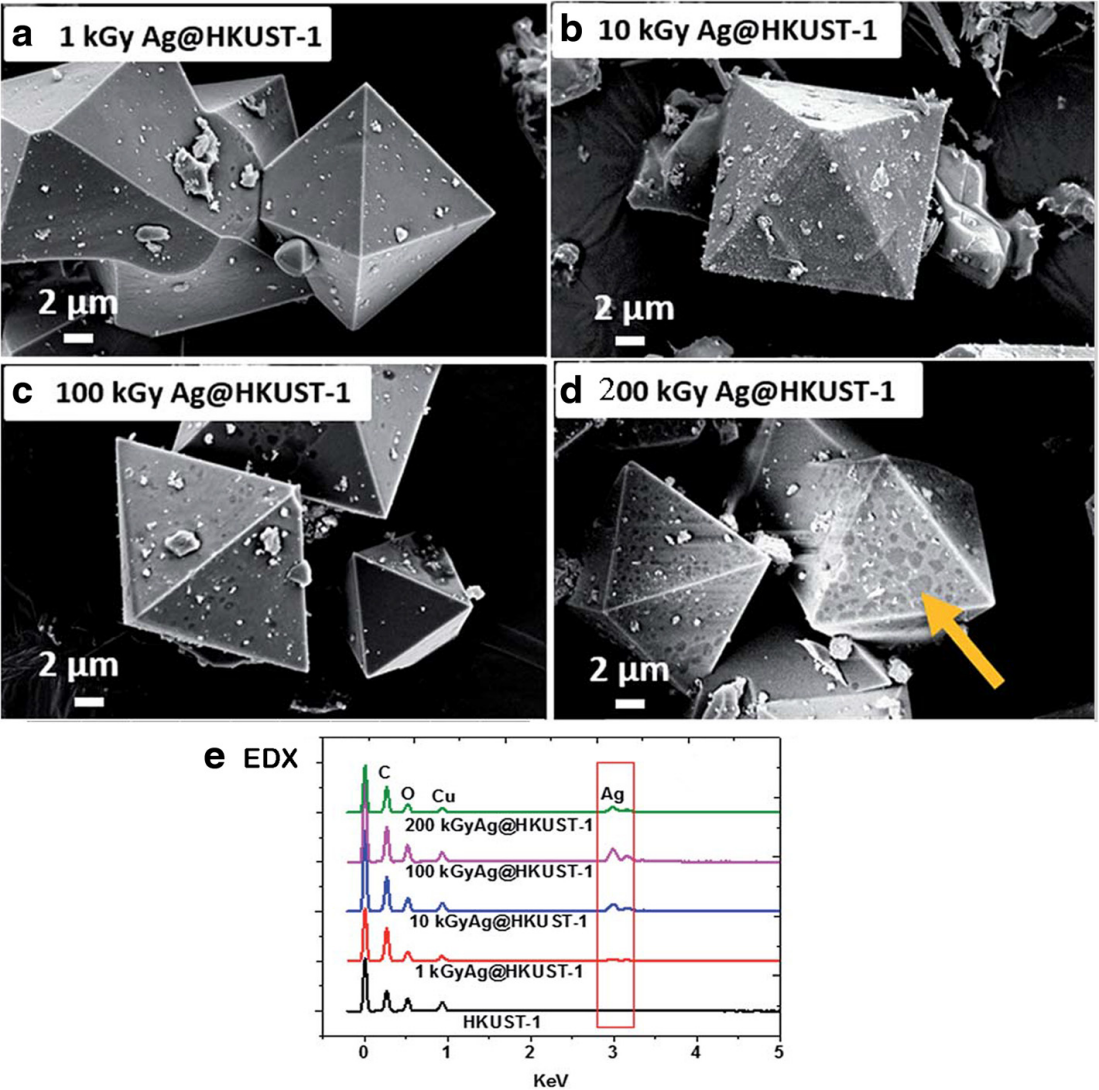
SEM images and EDX spectra of Ag@HKUST-1 crystals. SEM results of irradiated Ag@HKUST-1 crystals at different irradiation doses. **a** 1 kGy. **b** 10 kGy. **c** 100 kGy. **d** 200 kGy. **e** EDX spectra. Dark area at 200 kGy indicating localized damage at the surface [59]

The morphology of the irradiated Ag@HKUST-1 crystals at different gamma dose was investigated by using scanning electron microscopy (SEM) (Fig. 9). However, the overall morphology remained the same as that of the pristine HKUST-1 crystals but after irradiation, the crystals are covered with Ag nanoparticles which their intensity increased by irradiation dose. This result is clearly observed by the increase of Ag signal with radiation dose on elemental distribution of samples which was evaluated by EDX (Fig. 9e).

#### Effect of Metal Precursor

**Fig. 10:**
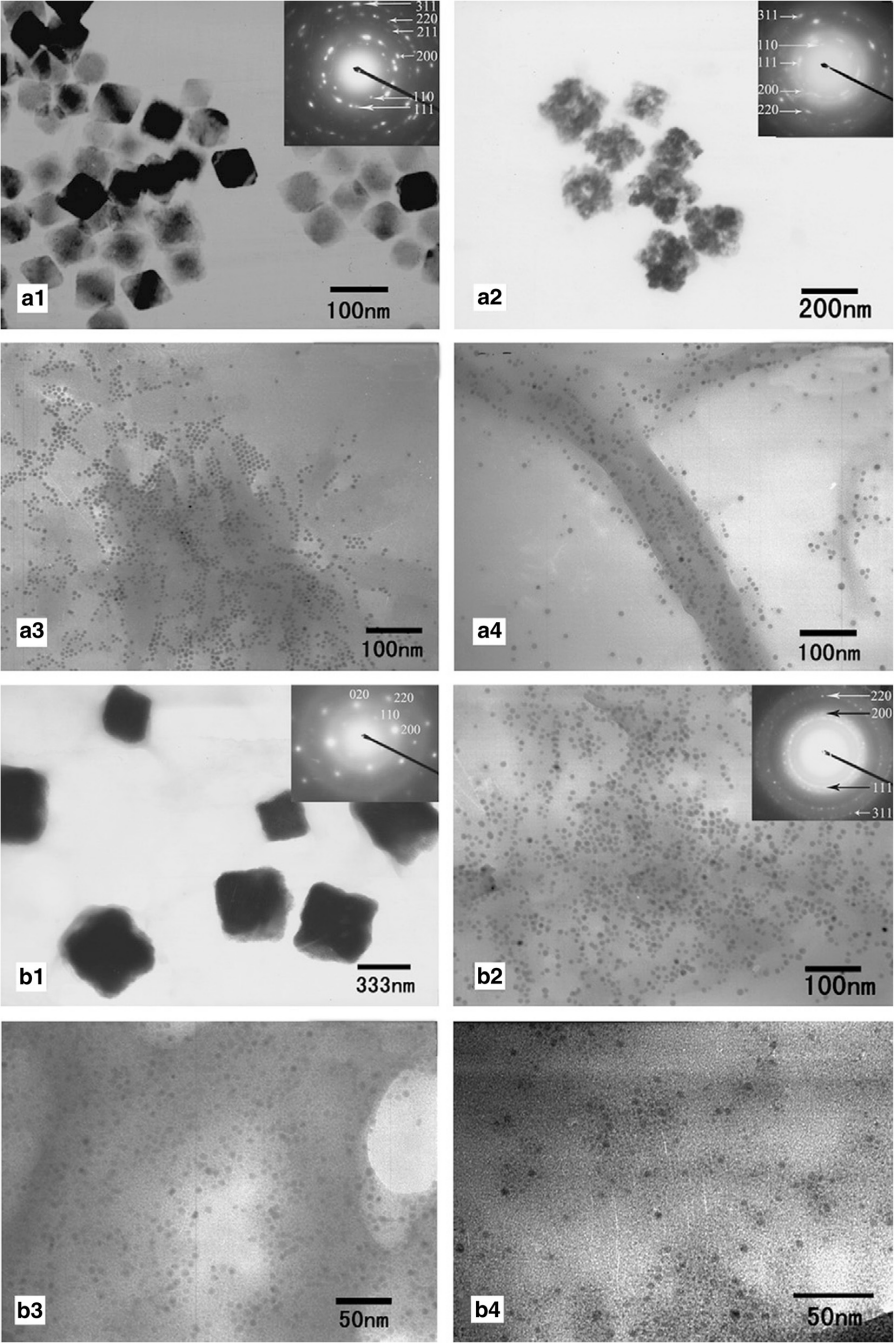
Different morphologies of the radiolytic synthesis of Cu-based nanoparticles. TEM images of irradiated Cu-based nanoparticles in: **a** Triton X-100-based and **b** Brij 56-based microemulsions (*ω* = 9.0) using different precursors: **a**_**1**_ **, b**_**1**_ Cu(NO_3_)_2_, **a**_**2**_ **, b**_**2**_ CuSO_4_, **a**_**3**_ **, b**_**3**_ CuCl_2_, **a**_**4**_ **, b**_**4**_ CuBr_2_. The *insets* show the SAED patterns of the corresponding products [63]

Different anions have shown visible effect on the morphology of metallic nanoparticles. Chen et al. [63] have reported the influence of type of anion on the radiolytic reduction of Cu^2+^ and the morphologies of the reduction products in the water-in-oil (W/O) microemulsions media with nonionic surfactants, i.e., Brij 30, Brij 56, or Triton X-100. In the Triton X-100-based and Brij 56-based microemulsions, where the *ω* value (molar ratio of water to surfactant) was fixed at 9.0, different morphologies of Cu nanoparticles have been synthesized by gamma irradiation with different precursors (Cu(NO_3_)_2_, CuSO_4_, CuCl_2_, and CuBr_2_) which are shown in Fig. 10. In gamma-irradiated microemulsion system, the scavenging of excess electrons, which were produced through the radiolysis of oil in the water, is the source of hydrated electrons (e^−^_aq_) [64].

Reaction between hydrated electrons (${\mathrm{e}}_{\mathrm{aq}}^{-}$) and NO_3_^−^ is much higher than others (Eq. 7). This reaction rate constant for ${\mathrm{SO}}_4^{2-}$, Cl^−^, and Br^−^ are <1.0 × 10^6^ L/mol s [65].

7 $$ {\mathrm{NO}}_3^{-}+{\mathrm{e}}_{\mathrm{aq}}^{-}\to {\mathrm{NO}}_3^{2-}\kern1em \mathrm{with}\ \mathrm{rate}\ \mathrm{constant}\ \mathrm{of}\ 9.7\times {10}^9\mathrm{L}/\mathrm{mol}\ \mathrm{s} $$

Consequently, in the Brij-56 system, NO_3_^−^ anions can scavenge the ${\mathrm{e}}_{\mathrm{aq}}^{-}$ easily and therefore, the amount of hydrated electrons decrease. In this case, the reduction product of Cu(NO_3_)_2_ is mostly in the form of square-shaped Cu_2_O, which is approved by the corresponding SAED analysis (inset, Fig. 10B_1_). On the other hand, the reduction products of CuSO_4_, CuCl_2_, and CuBr_2_ are in the form of Cu nanoparticles with quasi-spherical morphology.

The reduction products of CuSO_4_ in the Triton X-100 system are in the form of Cu_2_O nanoparticles which is not similar to the Brij-56 system due to the different mechanisms of reduction. Kapoor et al. [66] found that Cl^−^ and Br^−^ in this system can play the role of stabilizer for Cu nanoparticles and prevents the hydrolysis of Cu^+^ ions and generation of Cu_2_O nanoparticles.

#### Assembly of Preformed Nanoparticles

**Fig. 11:**
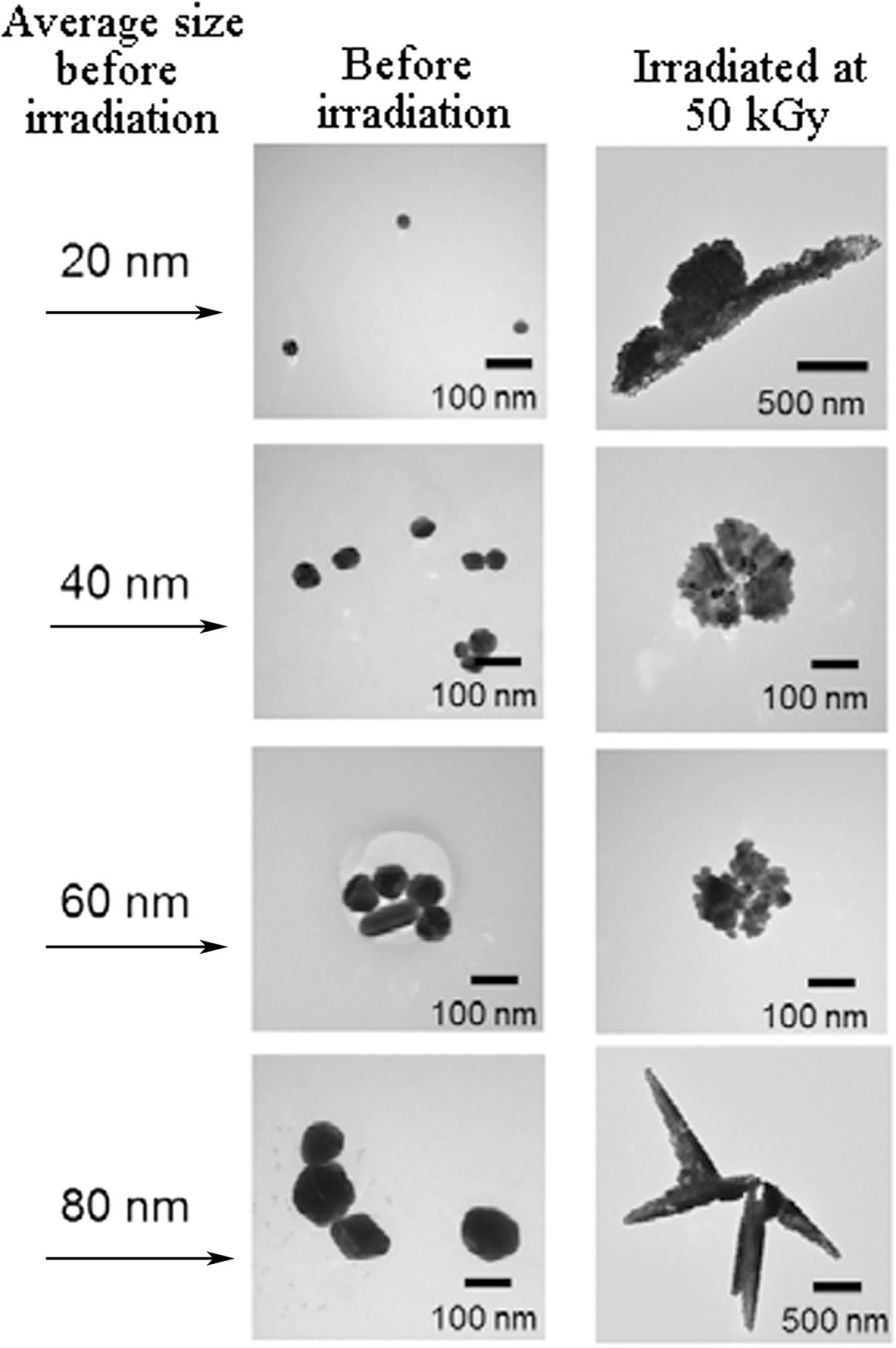
Representative TEM images of silver nanoparticles. TEM results of silver nanoparticles with average size of 20, 40, 60, and 80 nm before and after assembly process. (Adopted from [67])

The small metal nanoparticles which generated at the early stage of the reaction collide with each other due to their higher surface energy; therefore, they have pursued as building units for the formation of new structures with more complex shapes. Zheng et al. have hypothesized that the hydroxyl radical which produced during radiolysis of water can oxidize the presynthesized silver nanoparticles to form Ag^+^ ions [67]. In the next step, reducing agents in the solution reduced these ions to form Ag atoms, using the residual ablated particles as seed crystals. In the absence of sufficient stabilizer, coalescence process continues to reduce their high surface energy and therefore different types of morphology will be formed. However, growth based on the particle assembly is often predicted to generate nanoparticles with dendritic shapes, but the radiolytic assembly procedure is not a well controllable process, and different types of irregular shapes would be formed. Figure 11 shows different shapes of Ag nanoparticles before and after the radiolytic assembly process [67].

The modification and control of shape and size of this class of nanoparticles create new horizon for fascinating new applications. Here, we pointed out a brief illustration about the effect of morphology of radiolytically synthesized nanoparticles on optical behavior as an example, in order enlighten the importance of controlling the shape to reach specific properties.

### Optical Properties Enabled by Shape-Controlled Metal Nanoparticles

Shape-controlled metallic nanocrystals have been motivated for two main reasons. First, the new materials bring up the potential enhancements to industrial applications, and second is the prospect for new technology development. It is well known that the properties and performance of metal nanocrystals in a given application can be altering profoundly by changing the shape of the particles. Therefore, in this section, the modification of optical properties of shape-controlled metallic nanocrystals, which are synthesized by gamma radiation method, is mentioned. The main emphasis is on how these modified properties can be predictably adjusted to enhance performance or give rise to new applications. We believe that this section will promote further investigation into the properties of metal nanoparticles with controlled shape and also bring about the realization of new metal nanoparticles worth targeting synthetically.

Under the irradiation of light, the conduction electrons in a metal nanoparticles with sizes smaller than the wavelength of light are coupled to the electromagnetic field and driven by the alternating electric field to collectively oscillate in phase with it; this phenomenon is called LSPR [68]. Due to LSPR, metal nanoparticles can scatter and absorb certain wavelength of light under a resonant condition which enables noble metal nanoparticles (especially, Ag and Au) possess brilliant optical properties [1]. This particular wavelength of light, that metal nanoparticles interact with, can be modified by controlling the size and shape of the metal nanoparticles.

**Fig. 12:**
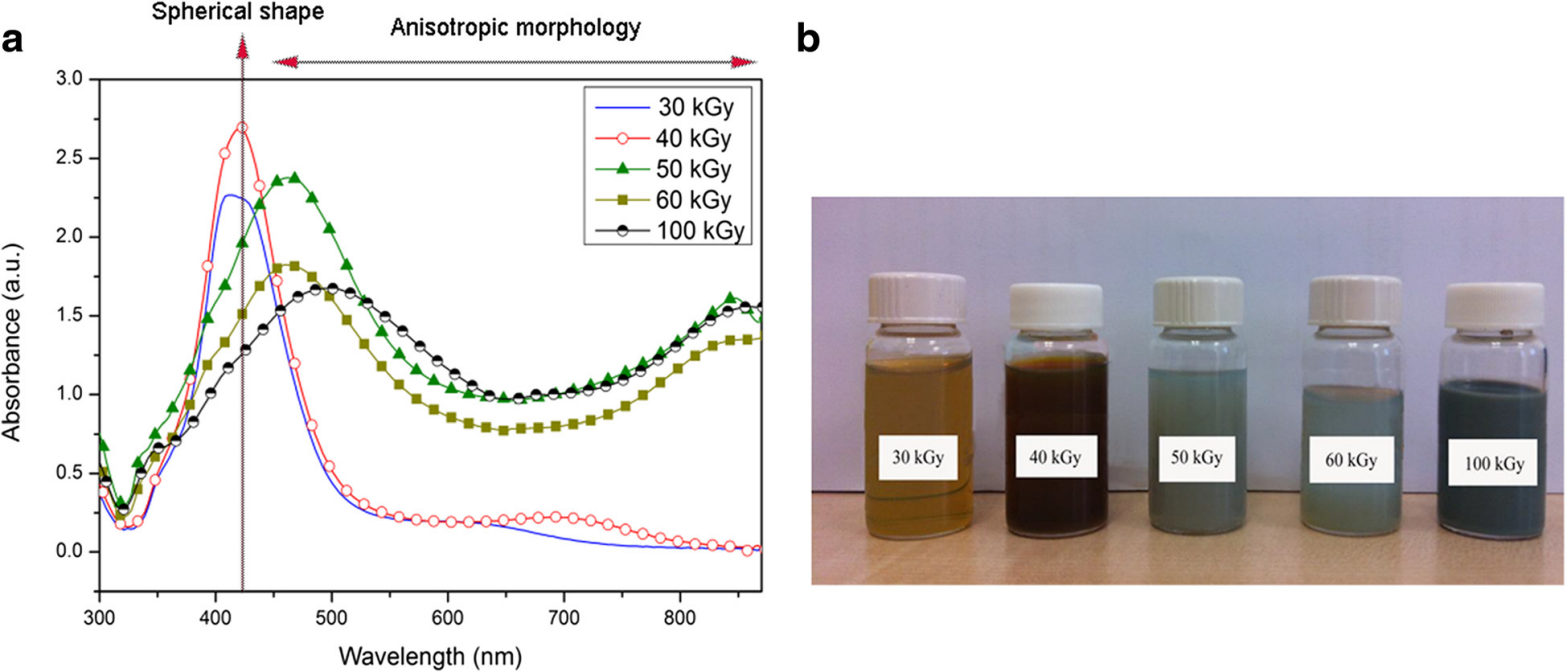
The changes in the UV-visible absorption and color of silver nanoparticles. **a** UV-visible absorption spectra of colloidal silver nanoparticles at different gamma doses. **b** Different colors of as-prepared silver nanoparticles. (Adopted from [55])

The changes in the color and UV-visible absorption of silver nanoparticles by variation in the morphology of irradiated samples were reported by Abedini et al. (Fig. 12) [55].

It is clearly observed from Fig. 12a that surface plasmon resonance of spherical silver in irradiated sample at 30 kGy shows symmetric intense peak at 425 nm. By increasing the gamma dose, the maximum plasmon absorption shifted toward longer wavelengths and new broad peak appears at long wavelength. This is as a result of increasing in particle size and shape transition of nanoparticles from spherical particles to triangular nanoplates. The peaks around 340, 453, and 850 nm are assigned to the out-of-plane quadrupole resonance, in-plane quadrupole resonance, and in-plane dipole plasmon resonance, respectively. At 100 kGy, because of an increase in edge length or decrease in the thickness of triangular nanoplates, a red-shift appears in the long wavelength in-plane dipole resonance [55].

**Fig. 13:**
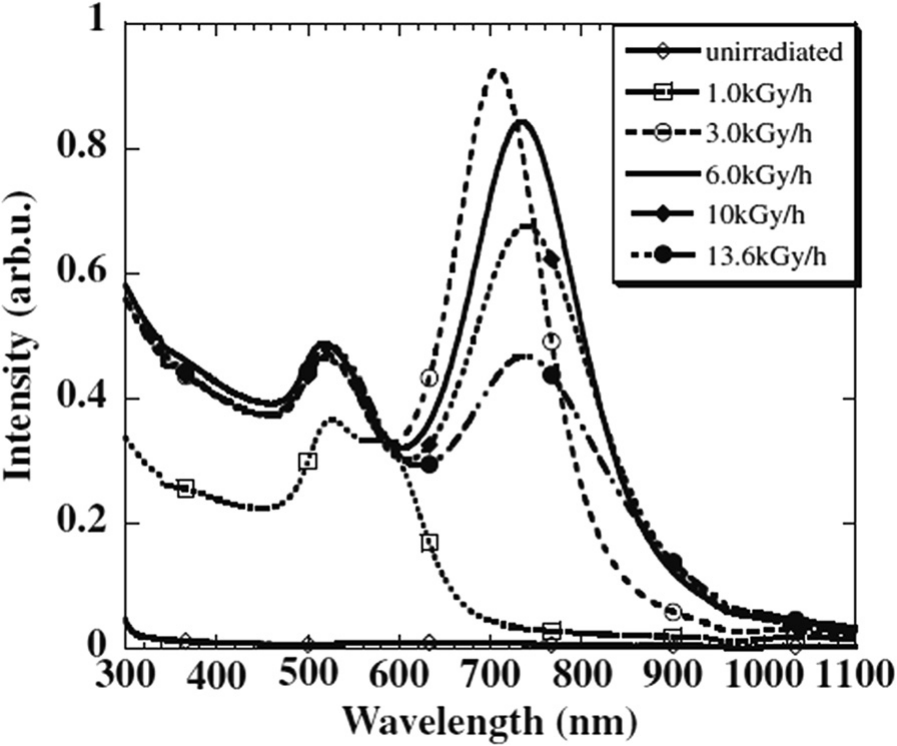
UV-visible absorption for different structures of Au nanoparticles. UV-visible absorption spectra for spherical and rod-shaped Au nanoparticles synthesized by gamma-ray irradiation with various dose rates. (Adopted from [57])

Okamoto et al. showed the shape dependency of UV-visible absorption of Au nanoparticles synthesized by gamma-ray irradiation with various dose rates (Fig. 13) [57]. As it was clearly observed from TEM images of these samples at Fig. 5, the formation of rod-shaped Au nanoparticles was dominant at higher dose rate. It is also observed that dose rate can modify the shape and size of Au nanorods. The absorption peak of spherical nanoparticles has been observed around 530 nm. The synthesis of rod-shaped Au nanoparticles at all radiation dose rates can be approved by appearance of another absorption peak around 700 nm. These two absorption peaks will be more separated due to changes in depolarization with increasing aspect ratio, and also, the longitudinal dipole begins to red-shift.

Since physical and chemical properties of nanomaterials strongly depend on their size and morphology, therefore, shape-controlled nanoparticles can introduce ideal candidates for devising new applications. For example, different metal nanostructures including nanospheres, nanotriangles, nanorods, and nanowires have been examined as SERS substrates to improve the sensitivity of conventional sensors. Yang et al. have studied on dependency of Ag nanoparticles’ shapes on SERS enhancement [69]. They have reported that, in comparison with spherical Ag nanoparticles, triangular nanoplates perform high activity at the substrates for the SERS detection, which is due to their sharp corners and edges. El-sayed et al. have also studied the impact of Au nanoparticles’ shape on the Raman enhancement [70]. They have reported that since the rod-shaped Au nanoparticles possess more (110) active facets than Au nanospheres, rod-shaped Au can strongly enhance the Raman signals [70].

**Fig. 14:**
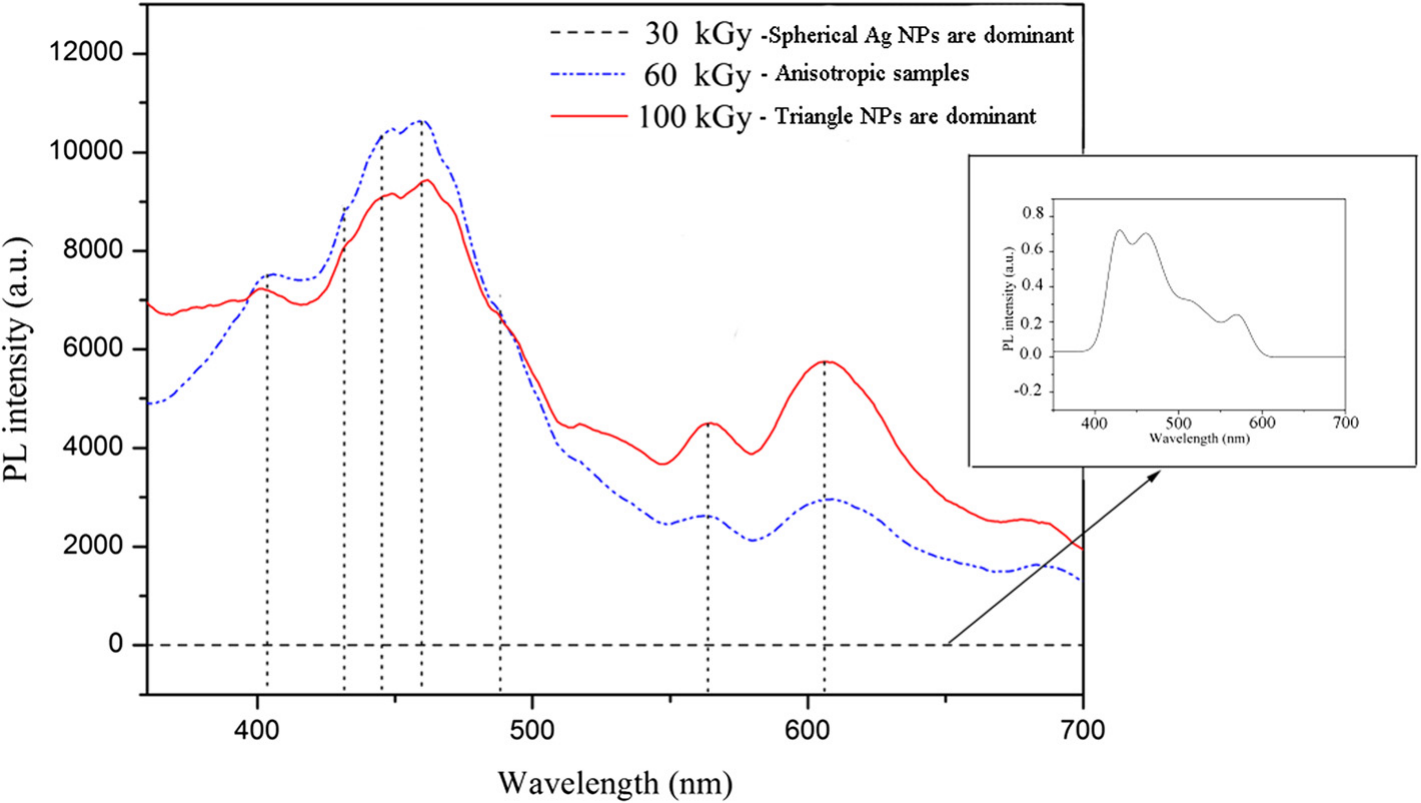
Photoluminescence results of Ag nanoparticles. Photoluminescence spectrum for Ag nanoparticles at different radiation dose. (Adopted from [55])

Another optical property that can be affected by shape transition of metal nanoparticles is photoemission spectrum. It has been reported that triangular colloidal silver nanoparticles are highly photoluminescent compare to their spherical shape [55]. Figure 14 shows the photoluminescence spectra of radiolytically synthesized Ag nanoparticles at different irradiation doses.

As it is clearly observed from Fig. 14, there are two groups of photoluminescence peaks located around 400–500 and 550–650 nm which have been enhanced by shape transition of Ag nanoparticles from spherical to triangular. The former group which is referred to the visible luminescence is due to interband transitions from “sp” electron states in the conduction band to holes states in the valence “d” band. The latter group corresponded to radiative decays of LSPR excitation in Ag nanoparticles and intraband “sp-sp” transitions [71, 72]. The dipolar and quadrupolar plasmon resonances of triangular Ag nanoparticles in irradiated samples at 60 and 100 kGy can make a strong local electric field which leads to the enhancement of the electric fields of the exciting (incoming) and emitting (outgoing) photons [55]. This enhanced luminescence has led to the use of shape-controlled noble metal nanoparticles especially Ag and Au in some applications, particularly cancer imaging, biological sensing, and the development of plasmonic solar cells [73, 74].

## Conclusions

We have introduced a number of useful strategies that can be tuned to control the radiolytic synthesis of noble metal nanoparticles with a specific shape in a solution phase. The nucleation process of seed and growth rate of different facets presents important role in determining the final shape of nanoparticles. Both nucleation and growth processes directly depend on coalescence and reduction rate which can be controlled by experimental parameters such as absorbed dose, dose rate, capping agents, and type of metal precursors which presented with several examples of recent researches. It is important to consider that the manipulation of the growth processes in the most attainable strategies is built for shape-controlled synthesis rather than the nucleation process. It is also illustrated how controlling the final shape can tune the optical properties of metal nanoparticles.
